# Micro-Expression Recognition Based on Dual-Stream Motion-Anchored Cross-Fusion Network

**DOI:** 10.3390/s26123628

**Published:** 2026-06-06

**Authors:** Junxian Li, Tian Li, Shucheng Huang, Gang Wang, Mingxing Li

**Affiliations:** 1 School of Information Engineering, Yangzhou Polytechnic University, Yangzhou 225009, China; junxianli@yzpc.edu.cn; 2 Suzhou Institute of Technology, Jiangsu University of Science and Technology, Suzhou 215699, China; 3 School of Computer, Jiangsu University of Science and Technology, Zhenjiang 212003, China; schuang@just.edu.cn; 4 School of Computing and Artificial Intelligence, Southwest Jiaotong University, Chengdu 611756, China; eachgood@my.swjtu.edu.cn; 5 JingJiang College, Jiangsu University, Zhenjiang 212013, China

**Keywords:** micro-expression recognition, optical flow, attention, emotion recognition

## Abstract

Micro-expression recognition (MER) remains a formidable challenge in affective computing due to the subtle, localized, and fleeting nature of facial muscle actuations. Conventional spatial-temporal networks are easily overwhelmed by static facial topologies, leading to feature representations that are heavily biased toward identity-specific noise. To address this, we propose the Motion-Anchored Cross-Modal Fusion Network (MACFN), a novel dual-stream ViT architecture that explicitly decouples and synergizes spatial appearance and optical flow dynamics. Specifically, we introduce a motion-anchored spatial attention module, which translates latent motion features into a sparse spatial probability mask. It acts as an enhancement gate, forcing the texture stream to bypass static backgrounds and attend to genuine ME-related regions. Furthermore, we design a cross-modal bilinear fusion module to capture the second-order interactions across modalities, mapping the coupled features into a discriminative semantic manifold. Extensive experiments conducted on the CASME II, SAMM, and SMIC databases under the rigorous leave-one-subject-out composite database evaluation protocol demonstrate that MACFN is effective and achieves competitive performance compared to several recent methods.

## 1. Introduction

Micro-expressions (MEs) are spontaneous and extremely brief facial muscle movements that typically occur when individuals attempt to conceal, repress, or disguise their genuine underlying emotions. Since their initial discovery in clinical psychiatry by Ekman and Friesen [1], MEs have been regarded as a physiological polygraph capable of revealing human subconscious emotions. Compared to macro-expressions common in daily communication, MEs exhibit two distinct physical properties: first, an exceedingly short duration (typically between 1/25 and 1/5 of a second); second, extremely low intensity, often manifesting solely as the subtle twitching or contraction of localized facial action units [2]. Due to their involuntary and objective nature, Micro-expression recognition (MER) has demonstrated immense application potential and irreplaceable scientific value in domains such as national security interrogation, clinical psychiatric diagnosis, advanced human–computer interaction, and public safety [3].

However, the inherent low-intensity and highly localized spatio-temporal characteristics of MEs impose significantly more severe computational challenges on MER tasks compared to traditional facial expression recognition. First, ME-induced pixel displacements are extremely subtle. These minute kinematic signals are easily overwhelmed by redundant static background noises, such as identity features, wrinkles, or lighting variations. Second, in natural unconstrained settings, humans inevitably exhibit respiratory fluctuations, involuntary blinks, or slight rigid head rotations. These macroscopic motion artifacts, which are irrelevant to emotional expression, often surpass the magnitude of the MEs themselves, severely disrupting the precise extraction of genuine non-rigid facial muscle deformations. Consequently, accurately isolating pure ME features under conditions of strong background noise and extremely low signal-to-noise ratios constitutes the core bottleneck in this field.

To address these challenges, early MER research primarily relied on hand-crafted spatio-temporal descriptors, such as local binary pattern from three orthogonal planes (LBP-TOP) [4], histogram of oriented optical flow (HOOF) [5], and main directional mean optical flow (MDMO) [6]. Although these methods achieved preliminary success on constrained datasets, hand-crafted features heavily depend on expert prior knowledge and lack generalized representation capabilities across complex illumination changes and inter-subject identity variations. In recent years, with the advent of deep learning, feature learning based on deep neural networks has progressively become the mainstream [7,8,9]. To capture both structural facial differences and instantaneous muscle deformations, explicitly decoupling spatial textures from motion dynamics has become a prevailing strategy.

Despite recent progress in dual-stream and spatio-temporal networks, existing MER methods remain constrained by three fundamental limitations: *(1) Representational domain redundancy and identity leakage:* Current methods typically perform parallel, indiscriminate feature extraction on the spatial facial stream and the optical flow stream [10,11]. However, ME occurrences are highly sparse and localized, meaning facial images contain vast background regions devoid of motion. Parallel processing not only incurs computational waste but also causes the network to overfit to static features. Consequently, the models become biased towards subject identity information, struggling to maintain cross-subject MER. *(2) Absence of long-range non-local dependency modeling:* The vast majority of baseline models employ convolutional neural networks (CNNs) as backbones. Constrained by the local receptive fields of convolutional kernels, CNNs fall short in capturing the synchronized, synergistic relationships between distant, non-adjacent facial muscles during an ME event (e.g., the simultaneous slight lowering of the brows and tightening of the lip corners). *(3) Shallowness and suboptimality of cross-modal fusion:* Traditional networks predominantly employ simple feature concatenation or element-wise addition at the terminal layers. Such linear combination mechanisms implicitly assume the independence of spatial and temporal modalities, completely ignoring the highly coupled joint probability distributions between spatial static textures and localized motion deformations.

To tackle the aforementioned issues, this paper proposes a novel Motion-Anchored Cross-Fusion Network (MACFN) aimed at decoupling and recombining the spatial textures and temporal dynamics of MEs. Specifically, to overcome the local perception flaws of CNNs, we construct a symmetric dual vision transformer (ViT) backbone to capture global facial muscle linkages. Regarding the input modalities, apex frame and optical flow derived from onset and apex frame are utilized as the inputs of dual ViT. At the cross-modal interaction level, we discard the conventional parallel process and propose the motion-anchored spatial attention (MASA) module. Using optical strain as a motion prior, MASA generates fine-grained spatial dynamic masks to prune static identity redundancy and explicitly purify the facial textural stream. Finally, we introduce a cross-modal bilinear fusion mechanism to thoroughly exploit the high-order statistical synergies between the filtered localized texture features and the optical flow motion representations.

The primary contributions of this paper are summarized as follows:

- We propose the Motion-Anchored Cross-Fusion Network (MACFN) featuring a symmetric Dual ViT backbone. It explicitly captures long-range non-local dependencies among distant facial muscles, overcoming the local receptive field constraints of CNNs.
- To resolve representational redundancy and identity leakage, we design the motion-anchored spatial attention (MASA) module. In addition, a cross-modal bilinear fusion mechanism with $L_{1}$ sparsity constraints is introduced to models the covariance synergies between purified textures and motion dynamics.
- Extensive experiments and evaluations under the leave-one-subject-out protocol demonstrate that the proposed MACFN is effective and superior to several recent methods.

## 2. Related Work

Since MEs are spontaneous, subtle, and fleeting facial movements, MER presents unique challenges in the computer vision community [12]. While early MER research relied heavily on hand-crafted features, the recent proliferation of deep learning has fundamentally reshaped the technical paradigm of this field.

### 2.1. Hand-Crafted Feature-Based MER

Before the widespread adoption of deep learning, MER predominantly depended on descriptors designed by domain experts to capture subtle spatio-temporal variations. LBP-TOP stands as one of the most representative traditional features, fusing spatial and temporal dimensions to encode fine-grained texture changes [4]. Subsequently, kinematic features such as optical flow [13] and histograms of oriented gradients (HOG) [14] were widely employed to quantify facial movements. However, hand-crafted descriptors inherently lack representational capacity and remain highly susceptible to illumination variations, inter-subject identity differences, and noise induced by involuntary head movements, severely limiting their efficacy in unconstrained and complex scenarios [2].

### 2.2. Deep Learning-Based MER

Compared to hand-crafted features, deep learning models can automatically learn high-level semantic representations in an end-to-end manner, thereby better capturing subtle and transient emotional shifts [15,16]. The current mainstream deep MER architectures primarily encompass the following directions: *(i) Spatio-Temporal Convolutional Networks:* To explicitly model motion along the temporal dimension, 3D-CNNs extend traditional spatial 2D convolutions into the temporal domain [17]. Studies demonstrate that on high-frame-rate datasets (e.g., CASME II [18] and SAMM [19]), 3D-CNNs exhibit superior capability in identifying low-intensity and repressed emotional manifestations compared to their 2D counterparts [20,21]. *(ii) Apex Frame or Optical Flow-based Networks:* Given the substantial information redundancy in ME video sequences, researchers have found that anchoring recognition on the apex frame, which is the moment of maximum facial muscle deformation, not only effectively leverages static image features but also yields classification performance superior to processing the entire sequence [8,13,22]. Consequently, many contemporary models adopt multi-stream architectures. These networks typically process the facial apex frame to extract spatial appearance features, and the dense optical flow (computed between the onset and apex frames) to capture temporal facial muscle variations, achieving better classification through simple feature fusion [7,23,24]. *(iii) ViT-based Networks:* Inspired by their exceptional capability in long-range dependency modeling, ViTs have been introduced to the MER domain [25,26]. By leveraging self-attention or cross-attention mechanisms, these networks can model the global dynamic relationships among various facial regions, allowing the model to focus on genuinely discriminative localized muscle deformations [17,27]. *(iv) Graph Convolutional Networks (GCNs) with Physiological Priors:* The manifestation of MEs originates from the coordinated activation of specific facial action units. To integrate this physiological topological prior, GCNs are extensively utilized in ME feature learning [23,28]. By defining facial landmarks or AUs as graph nodes and their anatomical correlations as edges, coupled with recurrent neural networks for spatio-temporal reasoning, GCN models demonstrate strong advantages in filtering out complex environmental interference [29,30].

### 2.3. Macro-to-Micro Transfer Learning

Unlike macro-expression corpora that contain millions of samples, ME datasets are notoriously difficult to elicit and annotate, resulting in small-scale collections with severe class imbalance [2]. This severe data paucity exposes deep classification networks to a significantly high risk of overfitting, making training from scratch highly unstable. To circumvent this bottleneck, transfer learning, leveraging large-scale macro-expression datasets to assist ME recognition, has emerged as a predominant optimization strategy. Since both macro and micro-expressions share the same underlying facial muscle anatomy, models pre-trained on massive macro-expression corpora (e.g., AffectNet [31] or RAF-DB [32]) can capture generalized structural priors, facial topology, and rich texture representations. Researchers have extensively explored cross-domain transfer techniques, utilizing these pre-trained weights to initialize MER networks, thereby substantially mitigating the data starvation problem and accelerating convergence [33,34]. However, direct or naive full fine-tuning of pre-trained models on ME datasets often leads to catastrophic forgetting of the robust structural priors [35]. Furthermore, recent works are dedicated to sophisticated domain adaptation mechanisms [36] to gracefully bridge this intensity gap while meticulously preserving the intrinsic subtlety of micro-expressions [37,38].

## 3. Methodology

### 3.1. Framework Overview

**Figure 1:**
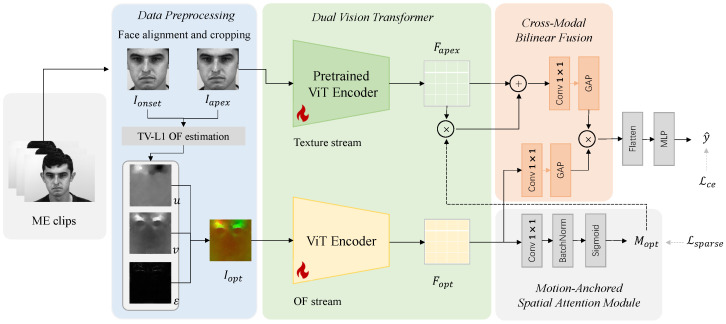
Overall pipeline of our proposed MACFN.

Figure 1 presents the overall pipeline of the proposed Motion-Anchored Cross-Fusion Network (MACFN), which comprises four consecutive stages. First, raw video sequences undergo rigorous preprocessing to perform face alignment, cropping the spatial apex frame alongside the temporal onset-to-apex optical flow. Second, a Dual-ViT backbone independently extracts high-dimensional representations from these two distinct modalities. Third, a motion-anchored spatial attention module leverages the optical flow features to aggressively prune redundant static information in the texture stream. Finally, a cross-modal bilinear fusion module models the cross-covariance between the filtered texture and the optical flow motion representations before the final classification.

### 3.2. Data Preprocessing

Given the subtle nature of micro-expressions, minor head translations or rotations can severely contaminate the motion features. Therefore, strict spatial normalization is a prerequisite.

#### 3.2.1. Face Alignment and Cropping

Let a raw ME clip be defined as a sequence of frames $V=\{I_{1},I_{2},\ldots ,I_{T}\}$, where *T* denotes the temporal length. We first perform rigid facial registration to eliminate translation and rotation variations that could severely corrupt subsequent motion estimation. Following [9], we utilize eye-center coordinates from the Dlib facial landmark detector [39] to compute an affine transformation matrix. This matrix explicitly warps all frames of a clip into a normalized coordinate space. The aligned frames are then cropped to $I_{i}\in R^{3\times H\times W}$ and $I_{apex}$ is selected to serve as the spatial texture modality, capturing the peak deformation state and contextual facial textures.

#### 3.2.2. TV-L1 Optical Flow Estimation

To explicitly decouple temporal dynamics from static appearance, we compute the dense optical flow field exclusively between the aligned $I_{onset}$ and $I_{apex}$. We adopt the Total Variation $L_{1}$ (TV-L1) algorithm [40], which preserves motion discontinuities at facial action unit boundaries. The optical flow field $w={\left(u,v\right)}^{T}$ is obtained by optimizing the following energy functional:

(1) $$E\left(w\right)=\int_{\Omega }\left|I_{apex}\left(x+w\right)-I_{onset}\left(x\right)\right|dx+\lambda \int_{\Omega }\left|\nabla w\right|dx,$$

where x denotes the spatial coordinates, and λ controls the piecewise smoothness, yielding horizontal (*u*) and vertical (*v*) displacements.

However, raw optical flow vectors represent absolute displacement and remain susceptible to subtle, involuntary rigid head movements that survive the alignment process. Because optical strain is computed as the spatial derivative of the flow field, it naturally cancels out uniform rigid translations. This operation effectively isolates non-rigid, localized muscle deformations from global motion noise. Since MEs are physiologically defined by these subtle, non-rigid contractions, optical strain provides a more precise and noise-resistant kinematic representation than raw flow alone. Since MEs are inherently defined by localized, non-rigid muscle deformations, we compute the optical strain to explicitly encode this physiological prior as [13] does. The strain tensor ϵ is derived from the spatial derivatives of the flow field:

(2) $$\epsilon_{xx}=\frac{\partial u}{\partial x},\;\epsilon_{yy}=\frac{\partial v}{\partial y},\;\epsilon_{xy}=\frac{1}{2}\left(\frac{\partial u}{\partial y}+\frac{\partial v}{\partial x}\right),$$

where $\epsilon_{xx}$ and $\epsilon_{yy}$ denote normal strains, and $\epsilon_{xy}$ is the shear strain. We compute the optical strain magnitude to represent the total local deformation intensity:

(3) $$\left|\epsilon \right|=\sqrt{\epsilon_{xx}^{2}+\epsilon_{yy}^{2}+\epsilon_{xy}^{2}}$$

By channel-wise stacking the absolute displacements and the deformation intensity, we construct a robust, three-channel kinematic modality $I_{opt}\in R^{3\times H\times W}$, where $I_{opt}=\left[u,v,|\epsilon |\right]$.

### 3.3. Dual Vision Transformer

Considering the subtle and spatially distributed nature of MEs, where disparate facial muscles frequently activate in a synchronized manner despite minimal visible displacement, capturing long-range spatial dependencies is critical. To this end, we adopt a dual vision transformer architecture. However, to bridge the inherent domain gaps between different input modalities, we formulate a carefully tailored asymmetric initialization strategy.

**Figure 2:**
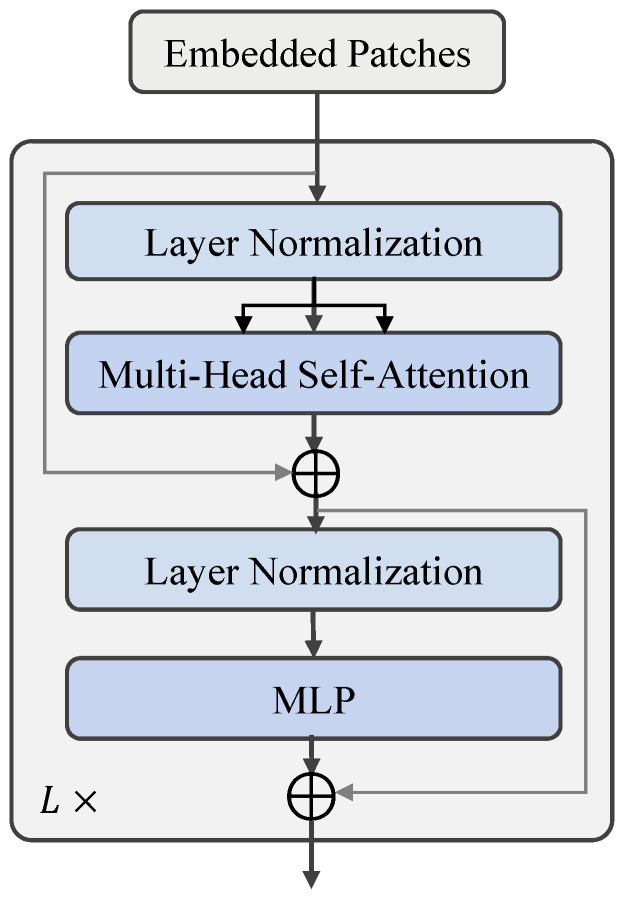
The internal structure diagram of the ViT encoder block utilized in our architecture.

For a given input modality $I\in \{I_{apex},I_{opt}\}$, the image is partitioned into $N=HW/P^{2}$ non-overlapping patches of size P×P. These patches are linearly projected to a *D*-dimensional latent space and prepended with a learnable class token $x_{cls}$. The token sequence is formulated as:

(4) $$X_{0}=\left[x_{cls}\|Ep_{1}\left\|\cdots \right\|Ep_{N}\right]+E_{pos},$$

where $E\in R^{(C\cdot P^{2})\times D}$ is the projection matrix, and $E_{pos}\in R^{(N+1)\times D}$ denotes the learnable 1D positional embeddings. Since the subsequent self-attention mechanism is inherently permutation-invariant, $E_{pos}$ is explicitly added to retain the spatial location information of each patch, which is essential for capturing spatially distributed muscle activations. The sequence is sequentially processed by *L* Transformer blocks. The architecture of the ViT encoder block is illustrated in Figure 2.

#### 3.3.1. Texture Stream

The spatial branch processes the apex frame $I_{apex}$. Due to the severely limited scale of MER datasets, training a ViT entirely from scratch inevitably leads to overfitting. Thus, we initialize this branch using weights pre-trained via Masked Autoencoders (MAE) on large-scale macro-expression corpora (i.e., AffectNet) [41,42]. Although the MAE objective provides robust, identity-agnostic structural priors, a significant domain gap in expression intensity remains: macro-expressions exhibit exaggerated deformations, whereas MEs are heavily suppressed.

To bridge the intensity domain gap between macro- and micro-expressions without succumbing to catastrophic forgetting, we bypass traditional full fine-tuning in favor of a hybrid parameter-efficient strategy. Specifically, we strictly freeze the parameter-intensive Attention and MLP projection matrices in the shallow and intermediate layers, thereby rigorously preserving the robust topological priors learned from macro-expressions. In these frozen stages, we only update the scale and shift parameters (γ,β) of the LayerNorms to align the feature distributions with the micro-expression domain. Simultaneously, we fine-tune the final two transformer blocks to capture high-level emotional semantics. This partial-tuning strategy effectively mitigates the risk of overfitting on small-scale MER datasets.

Upon extracting the output from the final block $X_{L}^{rgb}$, we discard the class token and reshape the sequence back into a 2D spatial grid $F_{apex}\in R^{D\times \frac{H}{P}\times \frac{W}{P}}$.

#### 3.3.2. Optical Flow Stream

The optical flow branch processes the composite motion tensor $I_{opt}=\left[u,v,|\epsilon |\right]$. Different from texture stream, we deliberately train this branch entirely from scratch. Imposing RGB-derived natural image weights on abstract motion fields and strain tensors leads to severe semantic misalignment and negative transfer, as optical flow fields are devoid of illumination, color, and structural facial textures. The patch embedding layer is randomly initialized using the Kaiming normal distribution. Given the scarcity of ME data, the optical flow branch employs ViT-Tiny, consistent with the texture stream, but comprises six blocks. This design forces the Transformer to discover temporal dynamic patterns and deformation correlations independently, yielding the optical flow motion representation $F_{opt}\in R^{D\times \frac{H}{P}\times \frac{W}{P}}$.

### 3.4. Motion-Anchored Spatial Attention

A fundamental limitation of directly fusing $F_{apex}$ and $F_{opt}$ is that the spatial feature map $F_{apex}$ encodes the entire facial topology, including expansive static regions that contribute solely to identity noise. We postulate that the motion feature $F_{opt}$ can act as a natural deterministic prior to isolate active muscle regions.

We formulate the motion-anchored spatial attention (MASA) module as an information bottleneck. The high-dimensional feature $F_{opt}$ of motion stream is projected into a single-channel spatial saliency mask $M_{opt}\in R^{1\times \frac{H}{P}\times \frac{W}{P}}$ via a localized 1 × 1 convolutional projection $W_{mask}\in R^{1\times D\times 1\times 1}$:

(5) $$M_{opt}=\sigma \left(B\left(W_{mask}*F_{opt}\right)\right),$$

where B(·) denotes batch normalization and σ(·) is the Sigmoid activation. This mask continuously evaluates the temporal activation probability of each spatial patch. By utilizing Hadamard product (⊙) broadcasting, we suppress the static structural noise in the texture domain:

(6) $${\tilde{F}}_{apex}=F_{apex}⊙M_{opt}+F_{apex}.$$

The residual connection guarantees that the gradient flow to the spatial branch remains stable and prevents the complete collapse of facial geometry during early training epochs.

### 3.5. Cross-Modal Bilinear Fusion

MEs are characterized by the joint probability distribution of spatial texture and facial localized dynamic. An ME action unit is defined not by isolated appearance or pure motion, but by their precise co-occurrence. Conventional first-order fusion techniques, such as simple feature concatenation or element-wise addition, inherently assume modality independence, merely aggregating isolated features without capturing their multiplicative interactions. To explicitly model this synergy, we propose a cross-modal bilinear fusion (CMBF) mechanism. Unlike first-order methods, CMBF calculates the outer product, acting as a second-order fusion strategy that explicitly models the full cross-covariance between spatial and temporal representations. Both ${\tilde{F}}_{apex}$ and $F_{opt}$ are projected to a compact latent space of dimension d=64. After applying Global Average Pooling to obtain instance-level vectors $v_{apex},v_{opt}\in R^{64}$, we compute their outer product to construct a full bilinear cross-covariance matrix $Z\in R^{64\times 64}$:

(7) $$Z=v_{apex}⊗v_{opt}=v_{apex}v_{opt}^{T}.$$

The matrix Z is flattened into a vector $z\in R^{4096}$. Because the outer product amplifies the variance of the features, we apply a power normalization (signed square-root) followed by $L_{2}$ normalization:

(8) $$z^{\prime }=\frac{\mathrm{sgn}\left(z\right)\sqrt{\left|z\right|}}{{∥\mathrm{sgn}\left(z\right)\sqrt{\left|z\right|}∥}_{2}+\epsilon }.$$

This highly compact representation $z^{\prime }$ is subsequently mapped to the emotion label space via a multi-layer perceptron (MLP). The MLP consists of a hidden layer with 512 units followed by a ReLU activation, a Dropout layer with a probability of p=0.5 to mitigate overfitting, and a final linear projection outputting *C* emotion logits.

### 3.6. Composite Optimization Objective

Prior to the loss computation, the $L_{2}$-normalized bilinear feature vector $z^{\prime }\in R^{d^{2}}$ is projected into the emotion label space via a MLP classifier. The MLP yields the logit vector $l\in R^{C}$, where *C* is the total number of emotion categories. The predicted probability distribution $\hat{y}\in R^{C}$ is subsequently obtained by applying the softmax function to the logits. For the *c*-th class, the probability is computed as:

(9) $${\hat{y}}_{c}=\frac{\exp(l_{c})}{\sum_{k=1}^{C}\exp\left(l_{k}\right)}.$$

The training of MACFN is supervised by a composite objective that tackles both the multi-class categorization accuracy and the intra-class spatial sparsity characteristic of MER datasets. For the primary classification task, we employ the standard cross-entropy loss to penalize the divergence between the predicted probability distribution and the one-hot encoded ground-truth label y:

(10) $$L_{ce}=-\sum_{c=1}^{C}y_{c}\log\left({\hat{y}}_{c}\right).$$

Furthermore, to strictly enforce the physiological assumption that micro-expressions are highly localized events driven by specific action units, we impose an $L_{1}$ sparsity regularization directly on the generated spatial mask $M_{opt}$. This constraint prevents the motion-anchored spatial attention (MASA) module from degenerating into a trivial identity mapping.

The total loss function $L_{total}$ is formulated as:

(11) $$L_{total}=L_{ce}+\lambda \frac{P^{2}}{HW}\sum_{i=1}^{H/P}\sum_{j=1}^{W/P}\left|M_{opt}^{\left(i,j\right)}\right|,$$

where the hyperparameter λ dictates the strength of the sparsity constraint, forcing the network to maintain an extremely tight attention focus on the active facial regions while suppressing the static structural noise.

## 4. Experiments

### 4.1. Datasets

To comprehensively evaluate the effectiveness of the proposed MACFN, we conduct extensive experiments on three spontaneous ME benchmark datasets: CASME II [18], SAMM [19], and SMIC [43].

- **CASME II:** This dataset contains 255 micro-expression video clips elicited from 26 participants. The videos were recorded using a high-speed camera at 200 frames per second (fps) with a spatial resolution of 280×340.
- **SAMM:** The SAMM dataset consists of 159 spontaneous micro-expression sequences from 29 subjects of 13 different ethnicities, recorded at a high frame rate of 200 fps and a high resolution of 2040×1088.
- **SMIC-HS:** We utilize the High-Speed version of the SMIC dataset, which includes 164 micro-expression clips from 16 subjects recorded at 100 fps.

Table 1 presents the detailed distribution of samples across the individual datasets and the merged Composite dataset.

### 4.2. Evaluation Metrics and Protocol

Following [44], we adopt the leave-one-subject-out (LOSO) cross-validation protocol. In each fold, all samples from one specific subject are held out for testing, while the remaining subjects are used for training. This protocol rigorously evaluates the subject-independent generalization capability of the model. In accordance with the MEGC2019 guidelines, we employ Unweighted F1-score (UF1) and Unweighted Average Recall (UAR) as our primary evaluation metrics. For a dataset with *C* classes, they are formulated as:

(12) $$UF1=\frac{1}{C}\sum_{c=1}^{C}\frac{2\times TP_{c}}{2\times TP_{c}+FP_{c}+FN_{c}},$$

(13) $$UAR=\frac{1}{C}\sum_{c=1}^{C}\frac{TP_{c}}{N_{c}},$$

where $TP_{c}$, $FP_{c}$, and $FN_{c}$ represent the true positives, false positives, and false negatives for the *c*-th class, respectively, and $N_{c}$ is the total number of ground-truth samples in class *c*.

### 4.3. Implementation Details

The proposed MACFN framework is implemented using PyTorch 2.11.0. All experiments are conducted on an RTX 4070Ti GPU. Unlike CASME II and SAMM, which include ground-truth apex frames, the SMIC dataset was released without them. Therefore, we apply the D&C-RoIs method [45] to detect apex frame in SMIC. The image are resized to 224 × 224 resolution and patch size is set to 16. The network is optimized end-to-end using the AdamW optimizer with a weight decay of $1\times 10^{-4}$. Both streams utilize ViT-Tiny configuration. Texture stream is initialized using weights pre-trained via MAE on large-scale macro-expression corpora, i.e., AffectNet [41,42]. We employ a layer-wise learning rate strategy: the texture stream utilizes a base learning rate of $5\times 10^{-5}$, whereas the optical flow stream and the fusion modules are trained with a higher learning rate of $1\times 10^{-3}$. We apply a linear warmup for the first 5 epochs to stabilize the initial gradients, followed by a cosine annealing learning rate scheduler. The model is trained for 100 epochs with a batch size of 16. The sparsity constraint weight λ in the composite objective function is set to 0.05.

### 4.4. Comparison Methods

To validate the superiority of the proposed MACFN, we systematically compare it against two categories of established micro-expression recognition methods:

- **Hand-crafted Feature Methods:** LBP-TOP [4], and Bi-WOOF [5]. These classical approaches rely on explicitly engineered spatio-temporal descriptors and serve as fundamental baselines.
- **Deep Learning-based Methods:** Standard multi-stream CNNs with optical flow as input, STSTNet [13], RCN-A [46], BDCNN [11], SMBANet [7], DSN-CASC [8], recent contrastive learning-based methods such as FRL-DGT [47], EMRNet [9] and fusion model like, LRNet [48], OFVIG-Net [30], and MPFNet [49]. This comparison highlights the structural advantages of our motion-anchored spatial attention and symmetric bilinear fusion over conventional cross-modal fusion strategies.

## 5. Results

### 5.1. Quantitative Results and Analysis

Table 2 presents a comprehensive performance comparison between our proposed MACFN and twelve prominent baseline methods on the composite dataset under the LOSO Composite Database Evaluation (CDE) protocol.

As shown in Table 2, our MACFN significantly outperforms all comparative methods on the overall composite database, achieving a new state-of-the-art UF1 of 0.8557 and UAR of 0.8588. Compared to recent transformer-based and multi-path fusion networks, such as OFVIG-Net and the second-best method MPFNet, MACFN yields substantial improvements of 2.37% and 1.18% in UF1 and UAR, respectively. This superiority on the composite benchmark thoroughly validates the robustness of our Dual-ViT architecture and the efficacy of the MASA mechanism in extracting domain-invariant emotional semantics across varying subjects and recording environments.

Beyond the overall metrics, the sub-dataset analyses reveal the specific strengths of MACFN. On the CASME II dataset, which is characterized by high frame rates and relatively high spatial resolution, MACFN reaches high, achieving a UF1 of 0.9603 and a UAR of 0.9554. It decisively surpasses the strong transformer-based competitor LRNet by margins of 3.49% (UF1) and 2.56% (UAR). This highlights the exceptional capability of our spatial-temporal decoupling strategy in leveraging high-fidelity optical flow to precisely pinpoint subtle facial muscle actuations. SAMM and SMIC datasets pose greater challenges due to more complex illumination variations, wider demographic diversity, and lower resolution (in SMIC). Nonetheless, MACFN secures the top-ranking UF1 scores on both datasets (0.8374 for SAMM and 0.7755 for SMIC). Although the latest MPFNet marginally edges out MACFN in terms of UAR on these two datasets (e.g., 0.8230 vs. 0.7979 on SAMM), its corresponding UF1 scores are relatively lower. In the context of severely imbalanced ME datasets, a high UAR coupled with a lower UF1 typically indicates a bias towards majority classes or an over-prediction tendency that sacrifices precision. These performance margins empirically validate our architectural distinctions over LRNet, OFVIG-Net, and MPFNet. While these prior works generally treat spatial and temporal modalities as independent parallel pathways and rely on first-order linear operations at the terminal layers, MACFN maintains the highest harmonic balance across all datasets. This quantitative gain directly proves the necessity of our two core designs: utilizing the kinematic modality as an active prior to prune static identity noise (via MASA), and replacing simple linear combinations with second-order bilinear fusion (CMBF) to explicitly model the highly coupled physiological synergy between purified textures and motion dynamics.

**Figure 3:**
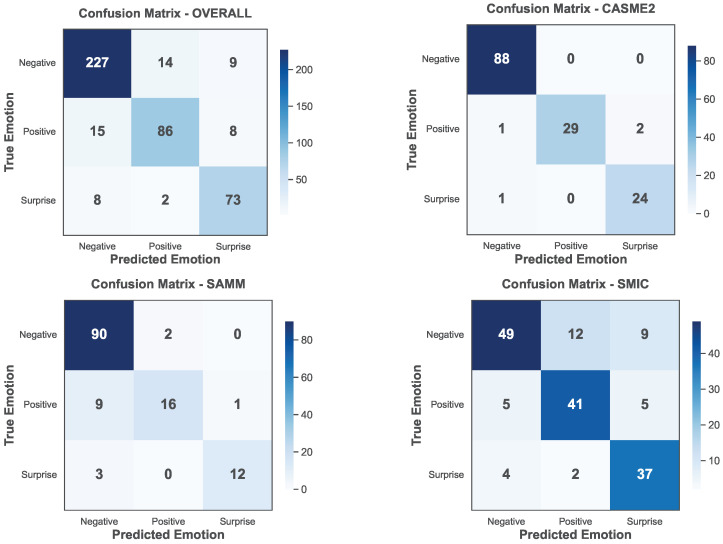
Confusion matrices for the overall dataset and each subset, evaluated on the combined dataset.

To further evaluate the fine-grained classification performance of the proposed method, Figure 3 presents the detailed confusion matrices for both the overall composite dataset and the three individual sub-datasets. Overall, the model demonstrates strong discriminative ability across the three primary emotion categories. The *Positive* and *Surprise* classes exhibit distinct diagonal dominance. However, the overall matrix reveals that the majority of cross-classifications occur between the *Negative* class and the other two categories. This phenomenon can be attributed to the fact that the *Negative* class in the composite dataset is intrinsically a super-class encompassing multiple distinct emotions (e.g., *Disgust*, *Anger*, *Sadness*, and *Fear*). Consequently, it contains a highly diverse and complex set of facial action units with significant intra-class variance, making its feature manifold much broader and harder to tightly bound.

Beyond the overall trends, the dataset-specific matrices reveal crucial insights into cross-domain variations and technical challenges. First, the model achieves near-perfect classification on the CASME II dataset, correctly identifying 100% of *Negative* samples and over 90% for both *Positive* and *Surprise*. This exceptional performance benefits from CASME II’s highly controlled laboratory illumination and its 200 fps high-speed recording, which ensure that optical strain accurately captures pure, high-fidelity muscle deformations. Second, in the SAMM dataset, while *Negative* and *Surprise* maintain high accuracy, there is a noticeable confusion where 9 *Positive* samples are misclassified as *Negative*. This is likely due to the extreme ethnic diversity in SAMM, where disparate facial bone structures and resting topologies introduce distinct projection biases into the positive muscle activations. Finally, SMIC proves to be the most challenging domain. The matrix shows a significant error margin where 12 *Negative* samples are misclassified as *Positive*, dropping the *Negative* accuracy to 70%. Unlike the other two datasets, SMIC-HS is recorded at 100 fps in a more unconstrained setting. The comparatively lower frame rate and spontaneous head movements can induce motion blur, severely disrupting the extraction of micro-kinematic boundaries between subtle negative and positive action units.

Nevertheless, despite these inherent cross-dataset challenges, the persistent diagonal dominance across all subsets confirms that MACFN effectively isolates genuine emotional representations from severe identity and environmental noise.

### 5.2. Ablation Study

#### 5.2.1. Module Ablation

To rigorously evaluate the individual contribution of each proposed module in our proposed MACFN, we conduct a comprehensive ablation study on the composite dataset under the CDE protocol. We define the baseline as a single-stream Vision Transformer (ViT-Tiny) and progressively integrate the optical flow stream, the MASA module, and the CMBF module. The quantitative ablation results are summarized in Table 3.

**Effectiveness of the Dual-Stream Architecture:** As observed in the first three rows of Table 3, relying solely on the texture stream yields the lowest performance. This is due to the subtle nature of MEs, where static texture features are easily overwhelmed by identity-specific information (e.g., gender, age, facial structure). The optical flow stream alone performs better, confirming that facial motion dynamics are the primary carriers of ME semantics. However, a naive dual-stream network using simple feature concatenation (Dual-ViT Naive) only achieves a UF1 of 0.7684. While it proves that appearance and motion are complementary, the limited improvement indicates that linear concatenation is insufficient to decouple and align the semantic gap between facial texture and motions.

**Effectiveness of the MASA Module:** The introduction of the MASA module yields a substantial performance leap. Compared to the naive dual-stream baseline, incorporating MASA (Dual-ViT + MASA) improves the UF1 from 0.7684 to 0.8145. This significant gain of 4.61% powerfully demonstrates the necessity of explicit spatial gating. By mapping high-dimensional optical flow into a bounded spatial probability mask using a sigmoid-activated information bottleneck, MASA effectively suppresses background identity noise. It forces the texture stream to focus exclusively on regions exhibiting active muscle strain, thereby preventing the network from overfitting to static facial topologies.

**Effectiveness of the CMBF:** To validate our fusion strategy, we replaced the simple concatenation with our CMBF while keeping other components identical. The model (Dual-ViT + CMBF) achieves a UF1 of 0.8211 without MASA, which is already competitive with state-of-the-art methods. When CMBF is combined with MASA to form the complete MACFN architecture, the performance reaches its peak. Unlike linear concatenation, which processes modalities independently, the outer product operation in SBF explicitly captures the second-order interactions across all channels of the texture and motion features. Followed by signed square-root and $L_{2}$ normalization, CMBF generates a highly discriminative, scale-invariant joint representation. The ultimate success of MACFN proves that both modules are mutually reinforcing, providing an optimal solution for capturing subtle variations inherent in MER.

**Module Synergy Analysis:** To clarify the individual impacts of the proposed modules, Table 3 reveals that CMBF is the dominant contributor to the overall performance gain. Integrating CMBF alone into the naive baseline yields a substantial absolute UF1 improvement of 5.27% (from 0.7684 to 0.8211), effectively surpassing the 4.61% gain provided by the MASA module alone. More importantly, the ultimate success of the full MACFN architecture (reaching a peak UF1 of 0.8557) stems from the explicit, sequential interaction between these two modules: purification followed by high-order synergy. Directly applying bilinear fusion to unpurified textures risks capturing spurious correlations between localized motions and global static identity noise. By deploying MASA as an upstream spatial gate, the network strictly bounds the textural features to active muscle regions. Consequently, CMBF computes the cross-covariance exclusively between pure structural deformations and genuine kinematic motions. This synergistic pipeline generates a highly discriminative, noise-free joint representation, proving both modules are mutually indispensable for effective MER.

#### 5.2.2. Comparative Analysis of Fusion Strategies

To validate the superiority of the CMBF over conventional fusion strategies, we conducted a comparative analysis. In this experiment, the MACFN backbone (Dual-ViT with MASA) was kept frozen, and the cross-modal interaction module was substituted with various fusion strategies. The quantitative comparison is detailed in Table 4.

As shown in Table 4, traditional first-order strategies, including element-wise addition and feature concatenation, yield sub-optimal performance. This performance bottleneck confirms that first-order operations implicitly assume statistical independence, merely aggregating isolated features without capturing their underlying synergies. Element-wise multiplication, a naive second-order operation, explicitly forces interaction between corresponding channel dimensions, resulting in a certain improvement. However, this stategy strictly confines interactions to spatially aligned features, neglecting modeling co-occurrence relationships in two modalities. In contrast, our proposed CMBF computes the full outer product, acting as a global second-order fusion mechanism. By capturing the complete cross-covariance matrix between each spatial texture and kinematic motion channel, CMBF mirrors that specific non-rigid strains must precisely co-occur with facial localized structural deformations. This alignment strategy allows CMBF to outperform standard concatenation by a substantial margin of 4.12% in UF1, firmly establishing its necessity and effectiveness in MER tasks.

### 5.3. Parameter Sensitivity Analysis

**Figure 4:**
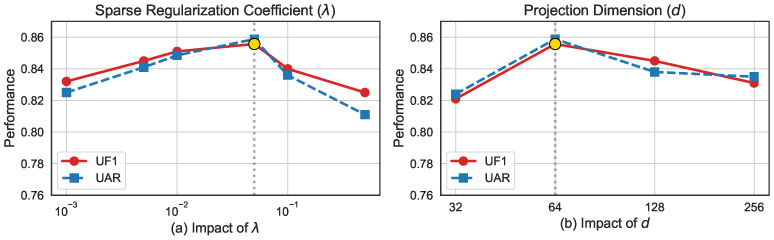
Parameter sensitivity analysis on the composite dataset. (**a**) Impact of the sparse regularization coefficient (λ). (**b**) Impact of the projection dimension (*d*) in CMBF. The optimal hyperparameters are marked in yellow.

In this subsection, we conduct parameter sensitivity analyses on two crucial hyperparameters: the sparse regularization coefficient (λ) for the MASA module and the projection dimension (*d*) for the CMBF. The corresponding performance variations on the composite dataset are illustrated in Figure 4.

**Impact of sparse regularization coefficient (λ):** The coefficient λ controls the intensity of the $L_{1}$ sparsity penalty applied to the MASA spatial probability mask. As depicted in Figure 4a, the model performance exhibits an inverted U-shape as λ increases. When λ is 0.0001, the sparsity constraint is too weak to suppress the identity-related static background, leading to sub-optimal recognition. As λ increases to an optimal range (0.01 to 0.05), both UF1 and UAR reach their peaks. In this state, the MASA module can filter out non-relevant facial static information better while sharply highlighting genuine localized activated areas. However, a continuously increasing λ overly aggressively regularizes the attention weights, mistakenly suppressing the subtle muscular dynamics inherent in micro-expressions and causing a decline in performance. Consequently, we set λ=0.05 as the default configuration across all experiments.

**Impact of projection dimension (d):** Before executing the CMBF, the 192-dimensional features from both the texture and optical flow streams are squeezed into a lower-dimensional space *d* using 1×1 convolutions. This projection dimension critically dictates the expressiveness and the parameter complexity of the CMBF module, which generates a d×d covariance matrix. Figure 4b demonstrates that a small dimension (d=32) restricts the representational capacity of the model, resulting in a loss of fine-grained emotion semantics. Conversely, excessively large dimensions (d=128 or d=256) explode the feature space to 16,384 or 65,536 dimensions, respectively. Given the limited sample size of micro-expression databases, such high-dimensional representations inevitably trigger the model overfitting. The sweet spot is observed at d=64, which strikes a suitable balance between preserving high-order interactive semantics and maintaining computational tractability.

### 5.4. Qualitative Results and Visualization

To intuitively understand the internal mechanisms and the feature representation capabilities of the proposed MACFN, we conduct qualitative evaluations from two perspectives: the spatial attention distribution generated by the MASA module and the high-dimensional feature manifold visualized via t-SNE.

**Figure 5:**
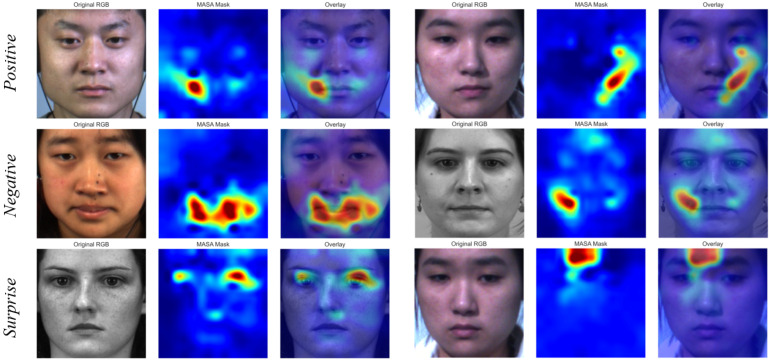
Visualization of the MASA maps. From left to right: the original apex frame, the generated spatial probability mask, and the overlay heatmap. The red regions indicate high attention weights, strictly corresponding to active facial areas.

**Visualization of MASA attention maps:** Figure 5 illustrates the spatial attention maps generated by the MASA module for several representative micro-expression samples with three distinct categories. The heatmaps explicitly reveal the network’s region of interest during the feature extraction phase. As observed in the overlay images, the high-response areas are strictly localized around specific facial muscle regions, such as the corners of the mouth (AU12, AU15) and the inner/outer extremities of the eyebrows (AU1, AU2, AU4). Conversely, static identity-related regions and the external background are effectively suppressed, manifesting as low-response areas. This precise spatial localization confirms that the MASA module, guided by the optical flow features, functions as a positive enhancement gate. It can force the facial texture stream to bypass redundant information and attend to the sparse, localized motions of MEs.

**Figure 6:**
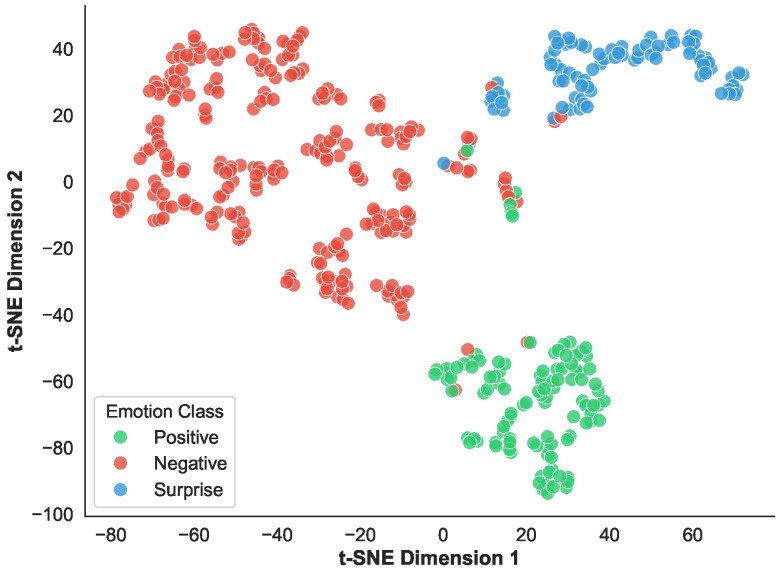
t-SNE visualization of the penultimate layer features extracted by MACFN on the composite dataset. The distinct clustering indicates high inter-class separability.

**t-SNE feature distribution:** To validate whether the proposed mothod yields discriminative semantic representations, we employ t-SNE to project the 512-dimensional features from the penultimate layer of the MLP classifier into a 2D space. As depicted in Figure 6, the feature distributions of the three emotion categories exhibit distinct topological boundaries. The *Positive* and *Surprise* samples form relatively compact and well-separated clusters. Although the *Negative* class exhibits a broader distribution spread, reflecting its inherent status as a composite super-class with significant intra-class variance, it remains globally separable from the other two categories with minimal overlapping margin.

## 6. Conclusions

In this paper, we presented the Motion-Anchored Cross-Fusion Network (MACFN) as an effective approach for MER, specifically aiming to mitigate the impact of identity interference and static background noise. By leveraging optical flow as a dynamic anchor, our proposed MASA module generates sparse spatial probability masks, which helps the network bypass redundant facial topologies and focus on localized muscle actuations. Additionally, CMBF module is utilized to capture the second-order interactions between spatial appearances and optical flow motion dynamics, forming a more discriminative joint representation. Experimental results on three datasets under the LOSO composite database evaluation protocol demonstrate the effectiveness of our approach. Compared to several existing baseline methods, MACFN provides competitive performance and stable improvements.

However, qualitative analyses indicate that classifying the composite *Negative* emotion remains a challenging task due to its significant intra-class variance. In future work, we plan to exploit framework by integrating self-supervised AU detection priors to better distinguish ambiguous emotional states.

## Figures and Tables

**Table 1:** The breakdown of ME samples with three categories.

Classes	SMIC	CASME II	SAMM	Composite
Negative	70	88	92	250
Positive	51	32	26	109
Surprise	43	25	15	83
Total size	164	145	133	442
Subjects	16	24	28	68
Ethnicities	3	1	13	–

**Table 2:** Experimental results on the composite dataset under CDE protocol. The first and second-best results are highlighted in bold and underlined, respectively.

Methods	Composite		CASME II		SAMM		SMIC	
	UF1	UAR	UF1	UAR	UF1	UAR	UF1	UAR
LBP-TOP [4]	0.5882	0.5785	0.7026	0.7426	0.3954	0.4102	0.5123	0.5280
Bi-WOOF [5]	0.6296	0.6227	0.7805	0.8026	0.5211	0.5139	0.5727	0.5826
STSTNet [13]	0.7353	0.7605	0.8382	0.8686	0.6588	0.6810	0.6801	0.7013
RCN-A [46]	0.7190	0.7432	0.8123	0.8512	0.6715	0.7601	0.6441	0.6326
BDCNN [11]	0.7470	0.7225	0.8740	0.8491	0.7572	0.7186	0.6317	0.6233
DSN-CASC [8]	0.7516	0.7607	0.9074	0.8995	0.6782	0.6897	0.6633	0.6727
FRL-DGT [47]	0.8120	0.8110	0.9190	0.9030	0.7720	0.7580	0.7430	0.7490
SMBANet [7]	0.7440	0.7459	0.8970	0.8920	0.6021	0.6068	0.6914	0.6934
LRNet [48]	0.8239	0.8300	0.9254	0.9298	0.7732	0.7851	0.7605	0.7639
EMRNet [9]	0.7468	0.7546	0.9074	0.8995	0.6782	0.6897	0.6509	0.6596
OFVIG-Net [30]	0.6720	0.6632	0.7129	0.7195	0.6066	0.5787	0.6435	0.6400
MPFNet [49]	0.8320	0.8470	0.8980	0.9070	0.7880	**0.8230**	0.7440	**0.7920**
MACFN (Ours)	**0.8557**	**0.8588**	**0.9603**	**0.9554**	**0.8374**	0.7979	**0.7755**	0.7881

**Table 3:** Ablation study of MACFN components on the composite dataset. “Concat” denotes simple feature concatenation.

Model Variant	Components		Module		Metrics	
	Apex Frame	Optical Flow	MASA	Fusion	UF1	UAR
Baseline (Apex Only)	✓			-	0.7212	0.7105
Baseline (Optical Flow Only)		✓		-	0.7538	0.7480
Dual-ViT (Naive)	✓	✓		Concat	0.7684	0.7592
Dual-ViT + MASA	✓	✓	✓	Concat	0.8145	0.8090
Dual-ViT + CMBF	✓	✓		CMBF	0.8211	0.8175
MACFN (Full)	✓	✓	✓	CMBF	0.8557	0.8588

**Table 4:** Comparative analysis of different cross-modal fusion strategies on MACFN.

Fusion Strategy	Type	UF1	UAR
Element-wise addition	first-order	0.8112	0.8065
Feature concatenation	first-order	0.8145	0.8090
Element-wise multiplication	second-order	0.8236	0.8210
CMBF	second-order	0.8557	0.8588

## Data Availability

Data will be made available on request.
